# The Efflux Inhibitor Phenylalanine-Arginine Beta-Naphthylamide (PAβN) Permeabilizes the Outer Membrane of Gram-Negative Bacteria

**DOI:** 10.1371/journal.pone.0060666

**Published:** 2013-03-27

**Authors:** Ryan P. Lamers, Joseph F. Cavallari, Lori L. Burrows

**Affiliations:** Department of Biochemistry and Biomedical Sciences, Michael G. DeGroote Institute for Infectious Disease Research, McMaster University, Hamilton, Ontario, Canada; University of Birmingham, United Kingdom

## Abstract

Active efflux of antimicrobial agents is a primary mechanism by which bacterial pathogens can become multidrug resistant. The combined use of efflux pump inhibitors (EPIs) with pump substrates is under exploration to overcome efflux-mediated multidrug resistance. Phenylalanine-arginine β-naphthylamide (PAβN) is a well-studied EPI that is routinely combined with fluoroquinolone antibiotics, but few studies have assessed its utility in combination with β-lactam antibiotics. The initial goal of this study was to assess the efficacy of β-lactams in combination with PAβN against the opportunistic pathogen, *Pseudomonas aeruginosa*. PAβN reduced the minimal inhibitory concentrations (MICs) of several β-lactam antibiotics against *P. aeruginosa*; however, the susceptibility changes were not due entirely to efflux inhibition. Upon PAβN treatment, intracellular levels of the chromosomally-encoded AmpC β-lactamase that inactivates β-lactam antibiotics were significantly reduced and AmpC levels in supernatants correspondingly increased, potentially due to permeabilization of the outer membrane. PAβN treatment caused a significant increase in uptake of 8-anilino-1-naphthylenesulfonic acid, a fluorescent hydrophobic probe, and sensitized *P. aeruginosa* to bulky antibiotics (e.g. vancomycin) that are normally incapable of crossing the outer membrane, as well as to detergent-like bile salts. Supplementation of growth media with magnesium to stabilize the outer membrane increased MICs in the presence of PAβN and restored resistance to vancomycin. Thus, PAβN permeabilizes bacterial membranes in a concentration-dependent manner at levels below those typically used in combination studies, and this additional mode of action should be considered when using PAβN as a control for efflux studies.

## Introduction

Multidrug resistant (MDR) Gram-negative pathogens are a global public health concern as therapeutic options for treating such infections are dwindling. *Pseudomonas aeruginosa* can exhibit resistance to nearly all antibiotics currently available and is a leading cause of hospital-acquired infections [1]. Multiple mechanisms contribute to antibiotic resistance in *P. aeruginosa*, including an inducible chromosomally-encoded AmpC β-lactamase, reduced outer membrane permeability, and active efflux mechanisms [2].

The AmpC β-lactamase inactivates β-lactam antibiotics which otherwise irreversibly inhibit the penicillin-binding proteins (PBPs) responsible for synthesis and maintenance of the bacterial cell wall [3]. Inhibition of PBPs by β-lactams [4], [5] causes increased accumulation of cell wall fragments that induce the expression of AmpC, which is exported to the periplasm where it hydrolyzes the antibiotics [3], [6]. Interestingly, inactivation of the non-essential PBP4, encoded by the *dacB* gene, also causes high-level AmpC expression in *P. aeruginosa* without β-lactam induction [7]. While this mechanism contributes to the high level β-lactam resistance for which *P. aeruginosa* is notorious, efflux-mediated antibiotic extrusion is an important aspect of the resistance phenotype [8].

Efflux-mediated antibiotic resistance in *P. aeruginosa* is conferred primarily by efflux pumps belonging to the resistance/nodulation/division (RND) superfamily that extrude a broad spectrum of antimicrobial compounds and other substrates [9]. The major RND efflux pumps of heightened clinical importance in *P. aeruginosa* are MexAB-OprM, MexCD-OprJ, MexEF-OprN, and MexXY-OprM, and differ in their expression patterns and substrate specificities [10]. Antibiotic substrates for efflux pumps include β-lactams, fluoroquinolones, and aminoglycosides [11]. MexAB-OprM is the constitutively expressed primary efflux pump in *P. aeruginosa* and responsible for the majority of efflux activity [12], [13]. Deletion of this pump renders *P. aeruginosa* highly susceptible to β-lactam antibiotics (even those strains overexpressing AmpC), while its overexpression increases antibiotic resistance [12], [14].

Given the contribution of efflux mechanisms to antibiotic resistance, efflux pump inhibitors (EPIs) have been widely sought as adjuvants to potentiate the activities of conventional antibiotics. Difficulties in identification of EPIs, however, have hampered their discovery and characterization, as controlling for non-efflux related effects can be challenging.

One of the best-studied EPIs is the peptidomimetic compound, phenylalanine-arginine β-naphthylamide (PAβN, also called MC-207,110) [15], [16]. PAβN was originally described in 1999 [16] and characterized further in 2001 [15] as a broad-spectrum efflux pump inhibitor, capable of significantly reducing fluoroquinolone resistance in *P. aeruginosa*. In addition to its efflux activity, PAβN was reported to permeabilize membranes at concentrations ≥16 µg/mL, but only in *P. aeruginosa* mutants deficient in the MexAB-OprM efflux pump [15]. A more recent study showed that as little as 4 µg/mL PAβN permeabilized wild type *E. coli* with potency similar to the antimicrobial peptide, Polymyxin B nonapeptide [17]. Both studies used increased access of small molecules to the periplasm as a measure of permeabilization. However, this alternative mode of action (MOA) is not widely considered in the literature as contributing to PAβN's antibiotic adjuvant activity.

While previous reports suggested that PAβN is not particularly effective at potentiating β-lactam activity [15], [18], [19], this question has not been adequately addressed. Because β-lactams are efflux pump substrates, inhibition of efflux should increase the potency of these drugs against *P. aeruginosa.* Here we report that PAβN enhances the potency of β-lactam antibiotics against wild type and AmpC-overexpressing strains of *P. aeruginosa*, as well as a strain deficient in the four major RND efflux pumps (MexAB-OprM, MexCD-OprJ, MexEF-OprN, and MexXY-OprM). Treatment with PAβN permeabilized the outer membrane of all *P. aeruginosa* strains tested, causing the release of AmpC β-lactamase into culture supernatants. Also, PAβN sensitized cells to vancomycin—an antimicrobial that is normally ineffective against *P. aeruginosa* because it cannot cross the outer membrane—and to bile salts. Our findings provide several lines of evidence showing that PAβN permeabilizes bacterial membranes, suggesting that its multiple modes of action need to be considered when it is used as a control in efflux studies.

## Materials and Methods

### Bacterial strains, plasmids, and mutants used in this study

Bacterial strains used in this study are listed in **Table 1**. The *P. aeruginosa dacB* mutant strain (*dacB::FRT*) was generated using a Flp-FRT recombination system, as previously described [20], with the FRT scar at nucleotide position 168. Briefly, *Escherichia coli* SM10 carrying the pEX18Ap-*dacB::GmFRT* disruption construct was incubated with *P. aeruginosa* strain PAO1, and mating mixtures were plated on *Pseudomonas* Isolation Agar (PIA) containing 100 µg/mL gentamicin to counter-select the donor. Gentamicin-resistant PAO1 colonies were plated on Luria-Bertani agar (LBA) containing no salt, 5% sucrose, and 100 µg/mL gentamicin to select for double recombinants that lost the *sacB*-expressing pEX18Ap suicide vector. Recombinants were screened for carbenicillin susceptibility and gentamicin resistance. For excision of the gentamicin resistance cassette, mutants were transformed with pFLP2 and selected on LBA containing 200 µg/mL carbenicillin. The pFLP2 plasmid was then cured on LBA containing 5% sucrose, without NaCl. PCR using *dacB*-specific primers was used to confirm gene disruption. The efflux-deficient *P. aeruginosa* strain (kindly provided by Dr. Keith Poole, Queen's University) was deleted of the genes encoding all four of the major RND efflux pumps – MexAB-OprM, MexCD-OprJ, MexEF-OprN, and MexXY-OprM [21].

**Table 1:** Bacterial strains used in this study.

Strain	Description	Reference
Wild type	*P. aeruginosa* PAO1 wild type	Prof. Keith Poole, Queen's University, Kingston, Ontario, Canada
*dacB*	PAO1 strain with FRT scar at nucleotide 168 of *dacB*	This study
Δefflux	PAO1 strain deficient in all four major RND efflux pumps (Δ*mexAB-oprM* Δ*mexCD-oprJ* Δ*mexEF-oprN* Δ*mexXY)*	Prof. Keith Poole, Queen's University, Kingston, Ontario, Canada

### Antibiotic susceptibility testing

Antibiotic susceptibility assays were performed using Etest strips (BioMérieux, France). Overnight bacterial cultures were sub-cultured 1∶50 in 5 mL Mueller-Hinton broth (MHB; Becton, Dickinson and Company, Mississauga, ON, Canada) with and without 1 mM MgSO_4_ containing 0, 10, 25, or 50 µg/mL PAβN and grown to logarithmic growth phase at 37°C and 200 rpm shaking. Cultures were standardized to OD_600 nm_ = 0.25 in MHB and 100 µL was spread on Mueller-Hinton agar (MHA) with and without 1 mM MgSO_4_ containing 0, 10, 25, or 50 µg/mL PAβN. Etest strips were overlaid and plates were incubated for 18 h at 37°C. Minimum inhibitory concentrations (MICs) were determined as the concentration at which the zone of inhibition intersected the Etest strip. All MICs were confirmed by three independent replicates. Two-fold or greater differences in MICs compared to control were considered significant.

### AmpC β-lactamase Western blot analyses

Overnight bacterial cultures were sub-cultured 1∶20 in 5 mL MHB containing 0, 10, 25, or 50 µg/mL PAβN and grown to an OD_600 nm_ = 0.8 at 37°C and 200 rpm. Cultures were then split 1∶1 in 5 mL MHB containing 0, 10, 25, or 50 µg/mL PAβN and incubated for an additional 2 h at 37°C and 200 rpm. Cell cultures were then standardized to an OD_600 nm_ = 0.6 and 1 mL was centrifuged at 2 300× g for 5 min. Cell pellets were resuspended in 100 µL of SDS sample buffer (0.3 M Tris-HCl, pH 6.8, SDS [6.7% w/v], glycerol [10% v/v], 2-mercaptoethanol [5.3% v/v] and bromophenol blue [0.2% w/v]), boiled for 10 min and stored at −20°C until immunoblotting. Supernatants from the same cultures were isolated and concentrated 20-times using Vivaspin 2, 10 kDa MWCO concentration columns (GE Healthcare Life Sciences, Montreal, QC, Canada). Samples were then combined with SDS sample buffer, boiled for 10 min and stored at −20°C until immunoblotting.

Immunoblotting was performed by first separating samples on 12.5% SDS-PAGE gels at 150 V for 90 min followed by transferring to nitrocellulose membrane at 225 mA for 60 min. Membranes were blocked using 5% skim milk in phosphate buffered saline (PBS; pH 7.0) at 37°C for 60 min followed by washing with PBS and incubation with α-AmpC primary antibody (1/10 000 dilution in PBS) (gift of Dr. Robert Bonomo, Case Western Reserve University; [22]) overnight at 4°C. Membranes were washed with PBS and incubated with α-rabbit secondary antibody conjugated to alkaline phosphatase (1/3000 dilution in PBS) for 1 h at 37°C followed by washing again with PBS. Membranes were developed in a solution containing 100 µL nitro-blue tetrazolium (NBT) and 100 µL 5-bromo-4-chloro-3-indolyl phosphate (BCIP) in 10 mL of alkaline phosphatase buffer (100 mM NaCl, 5 mM MgCl_2_, 100 mM Tris pH 9.5).

### Colony forming unit (CFU) assays

Overnight bacterial cultures were subcultured 1/20 in 5 mL MHB containing 0, 10, 25, or 50 µg/mL PAβN and grown at 37°C and 200 rpm to an OD_600 nm_ = 0.6. Cultures were then serially diluted in MHB containing appropriate PAβN concentrations followed by spreading 50 µL on MHA containing PAβN with and without 0.15% (w/v) No. 3 bile salts (Sigma-Aldrich, Oakville, ON, Canada). Plates were then incubated overnight at 37°C. The survival of *P. aeruginosa* was determined by enumerating CFUs from samples treated with both PAβN and bile salts as compared to control samples treated with either PAβN or bile salts alone. CFU assays were performed three times independently and statistical significance was assessed using a two-tailed Student's t-test. A p-value of ≤0.05 was considered statistically significant.

### Cell membrane permeability assays

To assess the integrity of bacterial cell membranes, the fluorescent probe, 8-anilino-1-naphthylenesulfonic acid (ANS; Sigma-Aldrich, Oakville, ON, Canada) was used. ANS is a neutrally charged, hydrophobic probe that fluoresces weakly in aqueous environments, but exhibits enhanced fluorescence in non-polar/hydrophobic environments [23]. Overnight bacterial cultures were sub-cultured 1∶20 in 5 mL MHB containing 0, 10, 25, or 50 µg/mL PAβN and grown to logarithmic growth phase at 37°C and 200 rpm. Cells were pelleted at 2 300× g for 5 min, standardized to OD_600 nm_ = 0.5, washed with 1 mL 5 mM sodium 4-(2-hydroxyethyl)piperazine-1-ethanesulfonic acid (HEPES; pH 7.2) (Sigma-Aldrich, Oakville, ON, Canada), and resuspended in 1 mL of the same buffer. For each sample a volume of 98 µL was added to triplicate wells of a white, clear-bottom, 96-well plate (Costar, Corning Inc., Corning, NY, USA). Two microliters of 3 mM ANS was added to each well and fluorescence was monitored using a Synergy™ HT multi-mode microplate reader (BioTek, Winooski, VT, USA) using an excitation wavelength of 375 nm and an emission wavelength of 510 nm. Relative fluorescence was calculated as the ratio of ANS fluorescence in untreated cells to matched, PAβN-treated cells. ANS assays were performed three times independently with each biological replicate consisting of three technical replicates. Statistical significance was assessed using a two-tailed Student's t-test and a p-value ≤0.05 was considered significant.

## Results

### PAβN reduces the intrinsic resistance of *P. aeruginosa* to β-lactam antibiotics

To assess whether PAβN potentiated the activity of β-lactam antibiotics against *P. aeruginosa*, including an intrinsically resistant mutant that overexpresses the chromosomally-encoded AmpC β-lactamase, minimum inhibitory concentrations (MICs) of three β-lactams were determined for wild type PAO1, AmpC-overexpressing (*dacB*) strains, and a strain deficient in the four major efflux pumps, MexAB-OprM, MexCD-OprJ, MexEF-OprN, and MexXY-OprM (Δefflux) in the presence and absence of PAβN. A PAβN concentration of 10 µg/mL had little effect on the MICs of β-lactams or the fluoroquinolone control, ciprofloxacin, in these strains (**Table 2**). However, PAβN concentrations of 25 or 50 µg/mL significantly reduced MICs for piperacillin, cefotaxime, ceftazidime, and ciprofloxacin in wild type and *dacB* strains (**Table 2**). The largest reduction in MIC was for the *dacB* strain treated with ceftazidime, where 25 or 50 µg/mL of PAβN caused an ∼42-fold decrease in resistance. In this strain, 25 or 50 µg/mL PAβN also reduced the MIC of piperacillin by 5-fold and 16-fold, respectively. PAβN treatment (10 µg/mL) caused a 2-fold reduction in piperacillin and ceftazidime MICs against the Δefflux strain, while 10 µg/mL PAβN reduced the MIC of cefotaxime 4-fold in this strain.

**Table 2:** Antibiotic susceptibilities of *P. aeruginosa* strains to piperacillin, cefotaxime, ceftazidime, and ciprofloxacin.

	Minimum Inhibitory Concentrations (µg/mL)^a^															
	Piperacillin				Cefotaxime				Ceftazidime				Ciprofloxacin			
	[PAβN] (µg/mL)				[PAβN] (µg/mL)				[PAβN] (µg/mL)				[PAβN] (µg/mL)			
Strain	0	10	25	50	0	10	25	50	0	10	25	50	0	10	25	50
PAO1	4 (6)	4 (4)	4 (4)	2 (4)	12 (16)	16 (12)	12 (12)	3 (12)	0.75 (1)	1 (1)	0.38 (1)	0.38 (1)	0.125	0.125	0.064	0.032
*dacB*	64 (96)	96 (96)	12 (48)	4 (12)	>256 (>256)	>256 (>256)	>256 (>256)	12 (24)	16 (24)	12 (24)	0.38 (8)	0.38 (2)	0.125	0.125	0.064	0.032
Δefflux	1 (1)	0.5 (0.5)	N/A	N/A	4 (3)	1 (1)	N/A	N/A	0.5 (0.75)	0.25 (0.38)	N/A	N/A	0.016	0.016	N/A	N/A

a MICs shown in parentheses were obtained with 1 mM MgSO_4_ in the assay.

N/A; efflux-deficient bacteria do not grow on Mueller-Hinton agar containing 25, or 50 µg/mL PAβN with or without 1 mM MgSO_4_.

Treatment with 25 or 50 µg/mL PAβN caused a 4-fold reduction in MICs of ciprofloxacin in the wild type and *dacB* strains. The Δefflux strain did not grow on MHA containing PAβN concentrations greater than 10 µg/mL, and this concentration had no effect on the MIC of ciprofloxacin.

### AmpC is released into culture supernatants upon PAβN treatment

**Figure 1:**
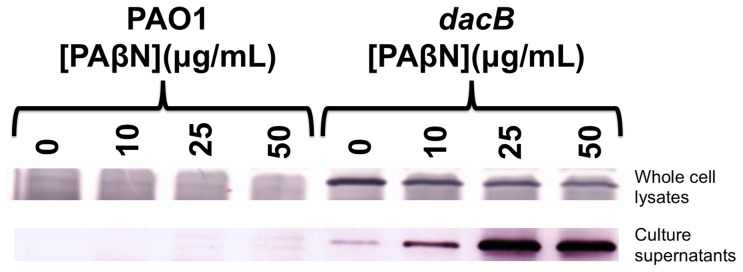
PAβN causes loss of periplasmic AmpC β-lactamase to culture supernatants. Treatment with PAβN reduces intracellular AmpC levels while increasing those in culture supernatants. Representative AmpC β-lactamase immunoblot comparing whole cell lysates and matched supernatants from wild type (PAO1) and AmpC overexpressing (*dacB*) *P. aeruginosa* strains treated with or without PAβN.

To determine whether PAβN reduced the MICs of β-lactams only by efflux inhibition, the expression and localization of AmpC was assessed in PAβN-treated bacteria. First, AmpC levels in whole cells were measured by Western blot with AmpC-specific antibodies. The levels of AmpC in whole cell lysates were reduced by ∼1.5-fold following PAβN treatment at concentrations as low as 10 µg/mL (**Figure 1** **)**. Further reductions in intracellular AmpC levels were observed as PAβN concentrations were increased to 25 (∼2-fold reduction) and 50 µg/mL (∼2.7-fold) (**Figure 1**). To determine whether PAβN reduced AmpC expression or released AmpC from the cells, culture supernatants were tested for the presence of AmpC. PAβN caused a concentration-dependent increase in extracellular AmpC (**Figure 1**), showing that while AmpC continued to be expressed, it was released into the culture supernatants from its normal location in the periplasm. These data suggested that PAβN was causing substantial outer membrane damage.

To address the possibility that PAβN was permeabilizing the cells, antibiotic susceptibility assays were repeated in the presence of 1 mM MgSO_4_ to stabilize the outer membrane. The MICs of all β-lactams were reduced with increasing PAβN concentrations—particularly in the *dacB* strain—although not to the same extent as the non-supplemented condition (**Table 2**). A PAβN concentration of 10 µg/mL reduced piperacillin MICs 1.5-fold in the wild type, but no additional reduction was observed with higher concentrations. For the *dacB* mutant, 10 µg/mL PAβN had no effect on β-lactam MICs; however, 25 and 50 µg/mL reduced the MIC of piperacillin 2-fold and 8-fold, respectively while 50 µg/mL PAβN reduced the MICs of cefotaxime >10-fold. In the same strain, 25 and 50 µg/mL PAβN also reduced the MICs of ceftazidime 3-fold and 12-fold, respectively. With Mg^++^ supplementation, 10 µg/mL PAβN reduced the MIC of piperacillin and ceftazidime 2-fold and that of cefotaxime 3-fold in the Δefflux strain. This strain did not grow on MHA with PAβN concentrations >10 µg/mL even with the addition of MgSO_4_. The release of AmpC to the supernatant by PAβN was reduced, but not eliminated, in the presence of MgSO_4_ (**Table 3**). The differences in MICs with and without Mg^++^ prompted further studies of PAβN's permeabilizing activity.

**Table 3:** Magnesium supplementation reduces the loss of intracellular AmpC upon PAβN treatment.

	Fold reduction in intracellular AmpC levels from *dacB* cells treated with PAβN, compared to untreated samples			
	0 µg/mL PAβN	10 µg/mL PAβN	25 µg/mL PAβN	50 µg/mL PAβN
Without Mg^++^	1.0	1.5	2.0	2.7
With Mg^++^	1.0	1.1	1.7	2.3

### PAβN treatment enhances *P. aeruginosa* susceptibility to bulky antibiotics

The susceptibility of *P. aeruginosa* strains to vancomycin—a bulky antibiotic that is normally incapable of crossing the outer membrane of Gram-negative bacteria—with and without PAβN was assessed. As shown in **Table 4**, all strains had MICs of >256 µg/mL in the presence of 0 or 10 µg/mL of PAβN; however, the vancomycin MIC was reduced to 96 µg/mL for the wild type and *dacB* strains treated with 25 µg/mL PAβN. Treatment with 50 µg/mL PAβN further sensitized these strains to vancomycin, with MICs for wild type and *dacB* strains of 32 µg/mL. Vancomycin sensitivity was abolished upon supplementation with 1 mM MgSO_4_ (**Table 4**).

**Table 4:** *P. aeruginosa* strains exhibit increased susceptibilities to vancomycin and erythromycin upon treatment with PAβN.

	Minimum Inhibitory Concentrations (µg/mL)^a^							
	Vancomycin				Erythromycin			
	[PAβN] (µg/mL)				[PAβN] (µg/mL)			
Strain	0	10	25	50	0	10	25	50
PAO1	>256 (>256)	>256 (>256)	96 (>256)	32 (>256)	64 (96)	64 (96)	16 (64)	4 (8)
*dacB*	>256 (>256)	>256 (>256)	96 (>256)	32 (>256)	64 (96)	64 (96)	24 (64)	6 (12)
Δefflux	>256 (>256)	>256 (>256)	N/A	N/A	3 (4)	1.5 (2)	N/A	N/A

a MICs shown in parentheses were obtained with 1 mM MgSO_4_ in the assay.

N/A; efflux-deficient bacteria do not grow on Mueller-Hinton agar containing 25, or 50 µg/mL PAβN with or without 1 mM MgSO_4_.

The MICs of wild type and *dacB* strains for another bulky antibiotic, erythromycin, a known substrate for RND efflux pumps [24], were reduced from 64 µg/mL for both strains at 10 µg/mL PAβN, to 16 and 24 µg/mL, respectively, upon treatment with 25 µg/mL PAβN. Treatment with 50 µg/mL PAβN further reduced erythromycin MICs to 4 and 6 µg/mL for wild type and *dacB* strains, respectively. Treatment of the Δefflux strain with 10 µg/mL PAβN reduced the MICs for erythromycin by 2-fold (from 3 µg/mL to 1.5 µg/mL). PAβN was less effective at reducing erythromycin MICs in PAO1 and *dacB* strains in the presence of Mg^++^ (**Table 4**). A PAβN concentration of 10 µg/mL also reduced erythromycin MICs in the Δefflux strain by 2-fold (from 4 µg/mL to 2 µg/mL) when Mg^++^ was present. Together these data suggest that PAβN's ability to potentiate antibiotic activity relies in part on its ability to destabilize the outer membrane.

### PAβN sensitizes *P. aeruginosa* to detergent-like compounds

**Figure 2:**
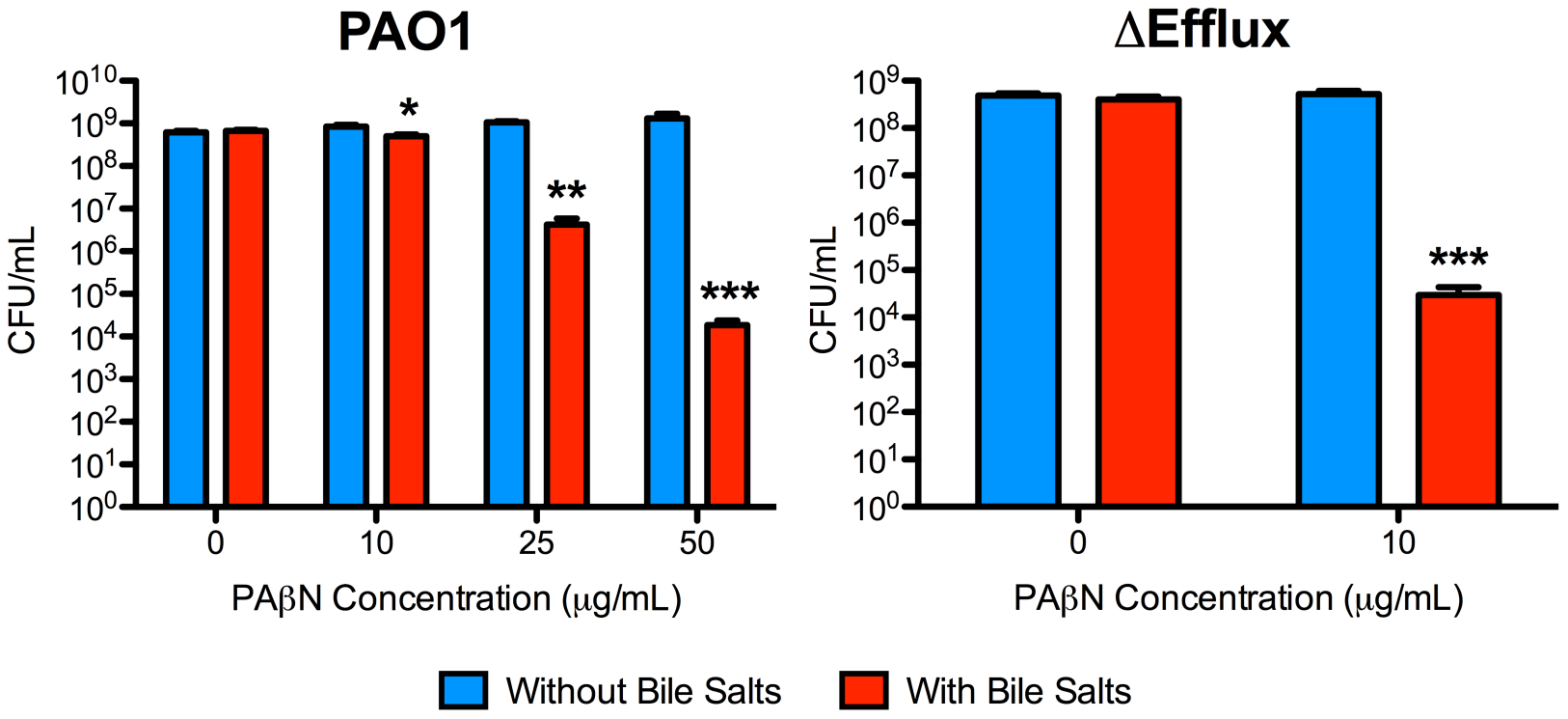
PAβN sensitizes *P. aeruginosa* to bile salts. Bacterial viability data from wild type (left) and efflux-deficient (right) *P. aeruginosa* strains grown in the presence of bile salts, with or without PAβN. As little as 10 µg/mL of PAβN significantly sensitized both strains to bile salts. Note that Δefflux bacteria do not grow on MHA containing >10 µg/mL PAβN. N = 3. Bars represent the means ± SEM. *p<0.05; **p<0.01; ***p<0.001.

Growth of wild type and Δefflux strains on agar containing detergent-like bile salts, to which Gram-negative bacteria are relatively resistant due to a combination of outer membrane impermeability and efflux [25], [26], in the presence or absence of PAβN was tested. Treatment with PAβN significantly reduced the viability of wild type bacteria in the presence of bile salts, with ∼3-log and ∼4.5-log reductions in viability at 25 µg/mL and 50 µg/mL PAβN, respectively (**Figure 2**). Viability of the Δefflux strain was reduced by ∼4.5-log in the presence of bile salts and 10 µg/mL PAβN, the highest concentration that could be tested.

### PAβN treatment increases ANS uptake

**Figure 3:**
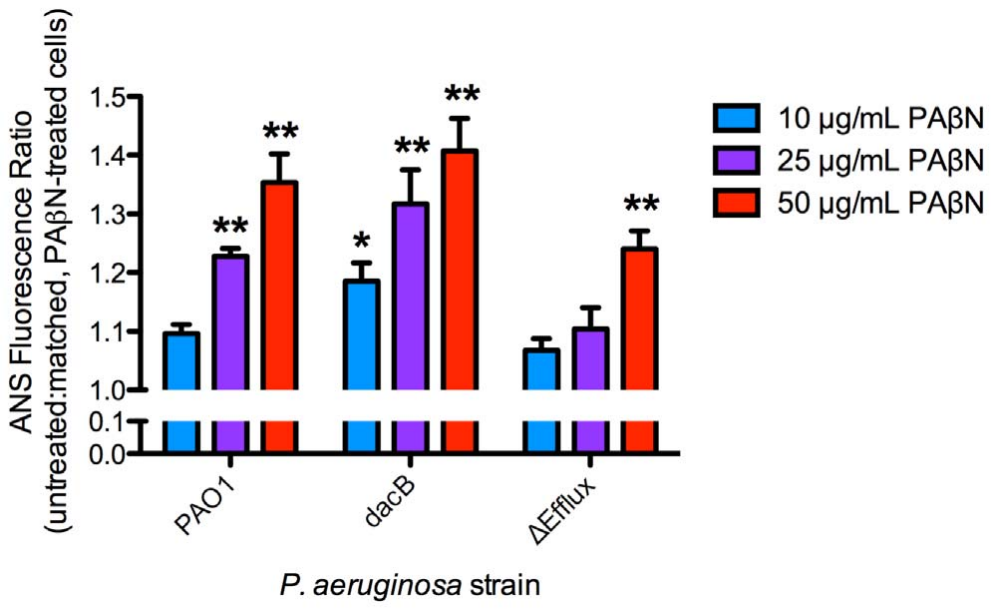
PAβN significantly increases ANS uptake. ANS fluorescence data, presented as a ratio of untreated and matched, PAβN-treated bacteria. N = 3. Bars represent the means ± SEM. *p<0.05; **p<0.01.

To assess the outer membrane integrity of bacteria treated with PAβN, we measured uptake of the fluorescent probe, ANS. At 10 µg/mL, PAβN treatment significantly increased ANS fluorescence in the *dacB* strain (p = 0.03) while PAβN concentrations of 25 µg/mL increased ANS fluorescence in both the wild type (23% increase; p<0.01) and *dacB* (32% increase; p<0.01) strains (**Figure 3**). Treatment with 50 µg/mL of PAβN increased fluorescence by 35% in wild type (p<0.01) and 40% in the *dacB* strain (p<0.01). Since the Δefflux mutant can grow in liquid media containing 50 µg/mL PAβN, this strain was included to assess whether loss of efflux pump activity in itself could account for the increase in outer membrane permeability. A significant increase in ANS fluorescence for this strain was observed with 50 µg/mL PAβN (p<0.01) (**Figure 3**).

Using the membrane potential-sensitive dye, DiSC_3_, PAβN was also tested for its ability to depolarize the inner membrane. Significant increases in DiSC_3_ fluorescence were observed following treatment with 10, 25, or 50 µg/mL PAβN (**Supplemental Figure S1**). Depolarization appeared to be transient, however, since no reduction in cell viability was observed at the completion of the assay (data not shown).

## Discussion

Drug efflux is a major contributor to multidrug resistance among bacterial pathogens [19]. Since blocking efflux could increase the intracellular concentrations and thus potency of currently available antibiotics, the discovery and development of efflux pump inhibitors (EPIs) is an important strategy. Unfortunately, studies of EPI activity can be complicated by non-efflux related effects.

Phenylalanine-arginine β-naphthylamide (PAβN) was originally identified as an EPI in 1999 as an efficient potentiator of fluoroquinolones (in particular, levofloxacin) in *P. aeruginosa* [15], [16]. Interestingly, it is not widely considered to potentiate β-lactam activity [18], [19], although such drugs are recognized efflux pump substrates and some synergy has been reported [15]. PAβN reduced β-lactam MICs for *P. aeruginosa* strains tested here, including those overexpressing AmpC. However, PAβN enhanced the activity of β-lactams differently in the absence and presence of Mg^++^, suggesting that increased susceptibility due only partly to efflux pump inhibition.

Our data suggest that PAβN permeabilizes both inner and outer membranes. AmpC is a soluble 43 kDa periplasmic enzyme and its accumulation in culture supernatants—and simultaneous depletion from its normally periplasmic location—upon PAβN treatment suggested that the outer membrane was compromised to the point where release of large, folded periplasmic proteins could occur, and this phenotype was only partly reversed by Mg^++^ supplementation. Mg^++^ dependent resistance to vancomycin in the presence of PAβN also indicated significant outer membrane permeabilization. Like most Gram-negative bacteria, *P. aeruginosa* is inherently resistant to vancomycin because the drug is normally incapable of crossing the outer membrane [27]. Similarly, erythromycin and bile salts (cholate and deoxycholate) are relatively ineffective against *P. aeruginosa*; however, PAβN increased the sensitivities of all strains tested, including the efflux-deficient mutant. These effects could be attributed partly to the expected inhibition of efflux; however, in the case of erythromycin, the fold reduction in MICs was different with and without Mg^++^, suggesting that membrane effects play a role. Increased susceptibility of the efflux-deficient strain to bile salts in the presence of PAβN may be enhanced by membrane permeabilization; however, it is also possible that additional (uncharacterized) efflux pumps encoded in the *P. aeruginosa* chromosome [28] might have a role in bile salt resistance. As a more direct measure of outer membrane permeabilization, PAβN treatment caused significant and dose-dependent increases in ANS fluorescence.

PAβN's ability to permeabilize membranes could be due to behavior as a cationic peptide. It is positively charged at physiological pH [15] and may displace divalent cations that bridge the phosphate groups on adjacent lipopolysaccharide core molecules in the outer membrane, causing instability. Membrane permeability might be caused by the loss of efflux activity; however, the Δefflux strain was completely resistant to vancomycin, suggesting that permeability is not increased in this mutant. However, permeability changes due to inhibition of other efflux pumps by PAβN treatment is still a possibility. AmpC leakage, increased susceptibility to β-lactams, and other pleiotropic effects have been reported in strains overexpressing the MexCD-OprJ efflux pump [29], [30], [31]. It is possible that efflux pump inhibition by PAβN causes compensatory increases in expression of other pumps that similarly alter membrane permeability.

Since RND-family efflux pumps are drug-proton antiporters that rely on inner membrane potential for their activity, inner membrane depolarization upon treatment with PAβN was assessed using the fluorescent dye, DiSC_3_. Under normal conditions, DiSC_3_ embeds in polarized membranes where its fluorescence is quenched; however, upon membrane depolarization, DiSC_3_ is released and fluoresces more intensely [32], [33]. Importantly, a significant but non-lethal perturbation of membrane potential was observed upon PAβN treatment. While it is not clear if transient depolarization is sufficient to disrupt efflux, it might partly account for previous reports of intracellular accumulation of pump substrates in *P. aeruginosa* upon PAβN treatment [15]. Inner membrane permeabilization could also explain the decreased viability of the efflux-deficient control strain when grown on solid media containing >10 µg/ml of PAβN. Since the compound is a pump substrate itself, presumably it accumulates to a greater extent in efflux-deficient strains with deleterious consequences for inner membrane integrity.

Previous reports suggested that PAβN may inhibit efflux in a competitive manner, by displacing other pump substrates [18], [34], [35], [36]. Co-crystallization of AcrB from *E. coli* with PAβN [37] showed binding to specific sites that could cause the exclusion of some substrates while allowing others to be bound and extruded [18], [35], [36], [38]. This finding is consistent with PAβN's ability to potentiate the activities of only a subset of efflux pump substrates. Our data suggest that it might potentiate the activity of non-pump substrates through its alternative MOA.

Given the widespread use of PAβN as an EPI in experimental studies, and the fact that it is often used as the gold standard to which new candidate EPIs are compared, it is important to highlight its alternative MOAs. The membrane permeabilizing activity of PAβN could be considered an asset, as it would promote its own entry into the cells where it can access its efflux pump targets. Small molecules such as PAβN that increase outer membrane permeability and/or impair drug efflux have excellent potential as antibiotic adjuvants that can reduce the effective doses of current drugs. They may also expand the range of usable antibiotics to those that so far have limited effectiveness against Gram-negative pathogens due to an inability to breach the outer membrane barrier [13], [35], [39].

## Supporting Information

Figure S1 **PAβN causes inner membrane depolarization in** ***P. aeruginosa*** **.** Wild type and efflux-deficient strains of *P. aeruginosa* were treated with increasing PAβN concentrations and assessed for inner membrane depolarization using the fluorescent dye, DiSC_3_. N = 3. Bars represent the means ± standard error. **p<0.01; ***p<0.001.
(PDF)
