# Supplementary material for: The Efflux Inhibitor Phenylalanine-Arginine Beta-Naphthylamide (PAβN) Permeabilizes the Outer Membrane of Gram-Negative Bacteria
Source: PLoS One. 2013 Mar 27;8(3):e60666. doi: 10.1371/journal.pone.0060666 (PMC3609863; doi:10.1371/journal.pone.0060666)
Supplement: Figure S1 — PAβN causes inner membrane depolarization in P. aeruginosa . Wild type and efflux-deficient strains of P. aeruginosa were treated with increasing PAβN concentrations and assessed for inner membrane depolarization using the fluorescent dye, DiSC3. N = 3. Bars represent the means ± standard error. **p<0.01; ***p<0.001. (PDF) [file pone.0060666.s001.pdf]

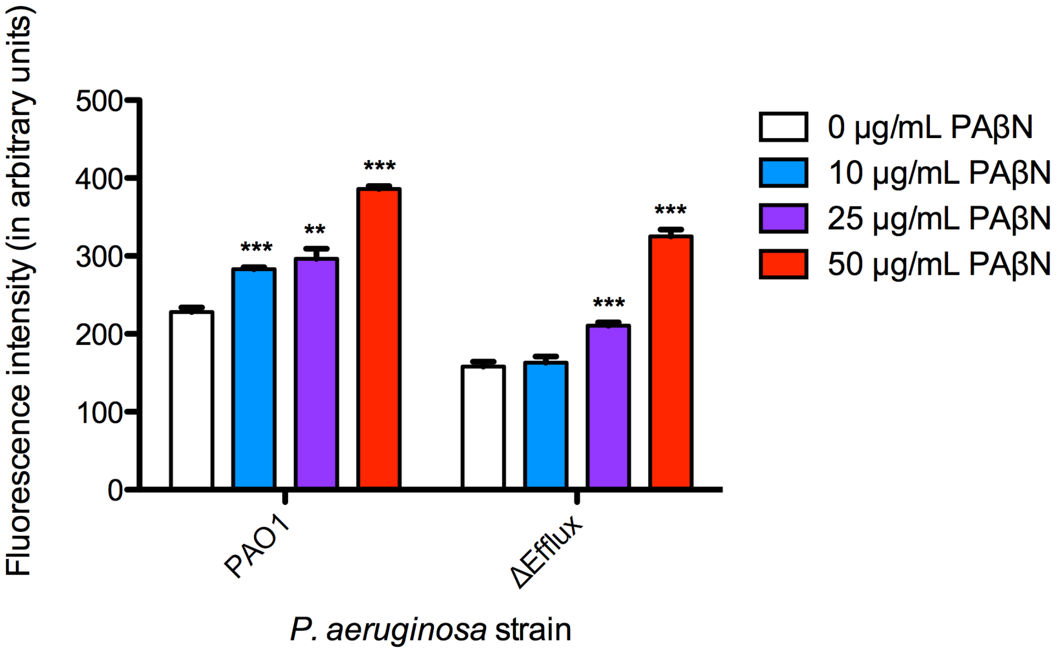

Supplemental Figure S1: PAβN causes inner membrane depolarization in *P. aeruginosa*. Wild type and efflux-deficient strains of *P. aeruginosa* were treated with increasing PAβN concentrations and assessed for inner membrane depolarization using the fluorescent dye, $DiSC_3$. N=3. Bars represent the means ± standard error. **p<0.01; ***p<0.001.

$DiSC_3$ Assay:

The $DiSC_3$ was performed as previously described [1,2]. Briefly, bacteria were grown to mid-logarithmic phase and washed and resuspended 5 mM sodium 4-(2-hydroxyethyl)piperazine-1-ethanesulfonic acid (HEPES; pH 7.2) (Sigma-Aldrich, Oakville, ON, Canada) containing 0.2 mM EDTA to an OD_{600nm}=0.05. Cells were incubated with 0.4 μM $DiSC_35$ (Sigma-Aldrich, Oakville, ON, Canada) for 45 min. to allow quenching. KCl (100 mM) was then added to equilibrate external and cytoplasmic K^+ concentrations. Samples (100 μL final) were added to triplicate wells of a white, clear-bottom, 96-well plate (Costar, Corning Inc., Corning, NY, USA) and appropriate PAβN

concentrations were added. Changes in fluorescence were recorded using a Synergy™ HT multi-mode microplate reader (BioTek, Winooski, VT, USA) using an excitation wavelength of 622 nm and an emission wavelength of 670 nm. Assays were performed three times independently and statistical significance was assessed using a two-tailed Student's t-test. A p-value ≤0.05 was considered significant.

References:

1. Zhang L, Dhillon P, Yan H, Farmer S, Hancock RE (2000) Interactions of bacterial cationic peptide antibiotics with outer and cytoplasmic membranes of *Pseudomonas aeruginosa*. Antimicrobial agents and chemotherapy 44: 3317-3321.
2. Qian CD, Wu XC, Teng Y, Zhao WP, Li O, et al. (2012) Battacin (Octapeptin B5), a new cyclic lipopeptide antibiotic from *Paenibacillus tianmuensis* active against multidrug-resistant Gram-negative bacteria. Antimicrobial agents and chemotherapy 56: 1458-1465.
